# Association of Perceived Built Environment Attributes with Objectively Measured Physical Activity in Community-Dwelling Ambulatory Patients with Stroke

**DOI:** 10.3390/ijerph16203908

**Published:** 2019-10-15

**Authors:** Masashi Kanai, Kazuhiro P. Izawa, Hiroki Kubo, Masafumi Nozoe, Kyoshi Mase, Mohammad Javad Koohsari, Koichiro Oka, Shinichi Shimada

**Affiliations:** 1 Department of Rehabilitation, Itami Kousei Neurosurgical Hospital, Itami 664-0028, Japan; kanaimasa07@gmail.com (M.K.); hiro.k16862@gmail.com (H.K.); 2 Department of Public Health, Kobe University Graduate School of Health Sciences, Kobe 654-0142, Japan; itamikousei1@yahoo.co.jp; 3 Cardiovascular Stroke Renal Project (CRP), Kobe 654-0142, Japan; koka@waseda.jp; 4 PREVENT Inc., Nagoya 461-0004, Japan; 5 Faculty of Sport Sciences, Waseda University, Tokorozawa 359-1192, Japan; javad.koohsari@baker.edu.au; 6 Department of Physical Therapy, Faculty of Nursing and Rehabilitation, Konan Women’s University, Kobe 658-0001, Japan; nozoe@konan-wu.ac.jp (M.N.); tkjwg268@yahoo.co.jp (K.M.); 7 Behavioural Epidemiology Laboratory, Baker Heart and Diabetes Institute, Melbourne, VIC 3004, Australia; 8 Melbourne School of Population and Global Health, The University of Melbourne, Melbourne, VIC 3010, Australia; 9 Department of Neurosurgery, Itami Kousei Neurosurgical Hospital, Itami 664-0028, Japan

**Keywords:** physical activity, urban design, cerebrovascular disease, stroke rehabilitation

## Abstract

There is little evidence on how perceptions of the built environment may influence physical activity among post-stroke patients. This study aimed to explore the associations between perceived built environment attributes and objectively measured physical activity outcomes in community-dwelling ambulatory patients with stroke. This cross-sectional study recruited patients who could walk outside without assistance. We assessed both objectively measured physical activity outcomes such as number of steps and duration of moderate-to-vigorous physical activity (MVPA) with an accelerometer and the patients’ perceived surrounding built environment with the International Physical Activity Questionnaire Environmental Module. Sixty-one patients (67.0 years old) were included. The multiple linear regression analysis showed significant associations of the presence of sidewalks (β = 0.274, *p* = 0.016) and access to recreational facilities (β = 0.284, *p* = 0.010) with the number of steps taken (adjusted R^2^ = 0.33). In contrast, no significant associations were found between perceived built environment attributes and MVPA. These findings may help to suggest an approach to promote appropriate physical activity in patients with stroke depending on their surrounding built environment.

## 1. Introduction

The promotion of physical activity can reduce the risk of all-cause mortality, cardiovascular disease, and type 2 diabetes [1,2,3]. A physically active lifestyle is important to maintain functional ability and independence in elderly adults [4]. Additionally, the promotion of physical activity is one key factor in preventing risk factors related to recurrent diseases [5,6]. Especially for patients with stroke, post-stroke physical activity relates not only to recurrent stroke [5] but also to all-cause mortality [7]. For example, Kono et al. reported that an initial target of at least approximately 6000 steps per day was appropriate to prevent the occurrence of new vascular events after stroke [5]. Another study conducted among stroke survivors reported that physical activity is inversely associated with all-cause mortality [7]. Therefore, promoting physical activity is one of the important strategies to prevent recurrent stroke.

Although the determination of physical activity is multifaceted and complex, ecological models emphasize the role played by the built environment in supporting an active lifestyle [8]. There are two methods of conceptualizing the relation of the built environment in relation to physical activity: subjective and objective assessment. The former relies on questionnaires to obtain residents’ perceptions about their surrounding built environment attributes [9,10,11,12], and the latter uses objective tools such as a geographic information system [13] and walk score [14] to measure environmental attributes. For patients with stroke, activity limitations or participation restrictions occur due to perceived barriers to engagement in physical activity [15]. Nicholson et al. indicated that environmental context and resources influence such perceived barriers [16]. However, Zhang et al. reported that patients with stroke did not consider attitudinal barriers as the dominant barriers to activity and participation [17]. The advantage of subjective assessment of built environment attributes for patients with stroke is that the built environment can be measured tailored to these patients. Nevertheless, there is little evidence regarding an association of post-stroke physical activity with perceived measures of built environment attributes in community-dwelling patients with stroke [16,17]. Therefore, this study aimed to determine associations between objectively measured physical activity and perceived measures of built environment attributes in community-dwelling ambulatory patients with stroke.

## 2. Materials and Methods

### 2.1. Study Design and Participants

Consecutive outpatients with stroke who consented to the measurement of their physical activity at Itami Kousei Neurosurgical Hospital from August 2016 to December 2018 were screened for inclusion in this cross-sectional study. The sample size used in the present study was determined based on the total number of patients at these durations.

The inclusion criteria included patients who could ambulate outside free of assistance regardless of the use of gait aids or orthotics. Exclusion criteria included patients living in a nursing home, those with dementia or moderate-to-severe aphasia as evaluated by their primary care physician, those with a modified Rankin Scale score [18] > 3 due to musculoskeletal disease, and those with severe cardiopulmonary disease or psychiatric disease based on evaluation of the patient’s medical records by a physical therapist.

This study was approved by the research ethics committee of Kobe University Graduate School of Health Sciences (no. 690). Informed consent was obtained from all patients.

### 2.2. Clinical Characteristics

We evaluated the patient characteristics of age, sex, body mass index (BMI), stroke subtypes (ischemic or hemorrhage), National Institutes of Stroke Scale (NIHSS) [19], time since stroke, comorbidities (presence of hypertension, diabetes mellitus, and dyslipidemia), walking speed, and whether a gait aid or orthotics were used. Patients were assessed with the NIHSS and a walking speed test by a physical therapist at the time of patient enrollment. Stroke severity was evaluated with the NIHSS score, which ranged from 0 to 42, with lower scores indicating mild severity [19]. Comfortable walking speed was determined from a 10 m walking test as 10 m/time required in seconds [20]. We used a stopwatch to time the patient’s walking time over a 10 m length of a 14 m walkway. We instructed the patients to walk at a comfortable walking speed and permitted the use of a walking aid or orthotics if these were normally used.

### 2.3. Objectively Measured Physical Activity

The primary outcome was physical activity values determined from the number of steps taken and duration of moderate-to-vigorous physical activity (MVPA). We used a Fitbit One 3-dimensional accelerometer (Fitbit, Inc., San Francisco, CA, USA) to measure the physical activity values. The Fitbit measures the number of steps taken, distance traveled, numbers of floors climbed, and calories burned, and sleep quality and has been used in previous studies of stroke patients [21,22,23]. After patient enrollment, the device was worn on the waist belt of all patients 24/h day for more than one week, except when bathing or changing clothes. The average number of steps taken daily was calculated over 7 consecutive days. MVPA was calculated as the sum of minutes of physical activity of greater than 3 metabolic equivalents in one day (min/day) and in one week (min/week) [23].

### 2.4. Perceived Built Environment Attributes

Perceived built environment attributes were evaluated using the International Physical Activity Questionnaire Environmental Module (IPAQ-E). The IPAQ-E contains 17 questions—7 core items, 4 recommended items, and 6 optional items. Consistent with several previous studies [9,10,11,12], we used the following 11 items, including both core and recommended items: residential density, access to shops, access to public transport, presence of sidewalks, presence of bike lanes, access to recreational facilities, crime safety, traffic safety, social environment, aesthetics, and household motor vehicles. These items refer to a surrounding built environment that a person could reach by walking 10 to 15 min from his or her residence. Nine of the 11 items (excluding residential density and household motor vehicles) include statements explaining neighborhood features considered related to physical activity that are responded to with four response options: strongly disagree, somewhat disagree, somewhat agree, and strongly agree. Responses for these environmental variables were classified into two categories: agreement (strongly agree and somewhat agree) or disagreement (somewhat disagree and strongly disagree). In terms of residential density, the choice of “detached single-family residences” formed a category indicating low residential density, whereas other forms of residence were included in a category indicating high residential density. Responses to the number of household motor vehicles were categorized as “none” and “one or more”.

### 2.5. Statistical Analysis

The results are shown as median (interquartile range) or as ordinal variables and counts (%) for categorical variables. Multiple linear regression analyses were used to determine the associations between objectively measured physical activity outcomes and perceived built environment attributes. The average number of steps and MVPA (min/day) were the dependent variables, and the independent variables included perceived built environment attributes correlating to objectively measured physical activity outcomes by Spearman correlation coefficient (*p* < 0.20). Covariates selected to adjust bias were age, sex, and NIHSS score. Because a previous study [24] indicated that walking speed played an important role in community walking activity after stroke, we also included walking speed as a covariate. A *p* value of < 0.05 was considered to indicate statistical significance. Statistical analyses were performed with IBM SPSS ver. 25.0 statistical software (IBM SPSS Japan, Inc., Tokyo, Japan).

## 3. Results

After three patients with invalid accelerometer and questionnaire data were excluded, a total of 61 participants were included in this study. The patients’ clinical characteristics are shown in Table 1. Most of the patients had suffered a mild stroke. However, 11 patients could walk with walking aids.

The average number of steps taken correlated significantly with the presence of sidewalks (ρ = 0.385, *p* = 0.002) and correlated with access to recreational facilities (ρ = 0.218, *p* = 0.091). MVPA correlated with residential density (ρ = −0.196, *p* = 0.131), presence of sidewalk (ρ = 0.241, *p* = 0.062), and social environment (ρ = 0.167, *p* = 0.198).

The results of the multiple linear regression analyses are shown in Table 2. After adjusting for all covariates, the presence of sidewalks (β = 0.274, *p* = 0.016) and access to recreational facilities (β = 0.284, *p* = 0.010) were significantly associated with the average number of steps taken. However, no perceived built environment attributes were significantly associated with MVPA.

## 4. Discussion

### 4.1. Key Findings

The findings of the present study show that the presence of sidewalk and access to recreational facilities were significantly associated with the average number of steps taken by community-dwelling ambulatory patients with stroke. However, no associations were observed between these perceived built environment attributes and MVPA.

### 4.2. Post-Stroke Physical Activity

In the present study, the average number of steps taken was 5556.7 steps/day, and MVPA was 13.9 min/day and 97.0 min/week. Although post-stroke physical activity depends on the severity of the stroke, a meta-analysis produced an estimate of 4355.2 steps/day (*n* = 315, 11 studies) among post-stroke patients [25]. A systematic review reported that patients with stroke took an average of 5535 steps/day (*n* = 406, 10 studies) in the subacute stroke phase and 4078 steps (*n* = 1280, 32 studies) in the chronic stroke phase [26]. In the present study, the subacute phase was defined as 14 days to 6 months after the stroke, and the time since stroke was 4.0 (interquartile range: 3.5–5.2) months. Thus, the average number of steps taken in the present study was equivalent to those taken in the subacute phase of these other studies [25,26].

The patients in the present study could not achieve the recommended level of MVPA of 150 min/week of moderately intense activity or 75 min/week of vigorously intense activity [27]. These recommended levels of physical activity described in other studies [28,29,30,31] may be hard for patients with stroke to achieve. Walking speed is one of the key factors of physical activity and the ability to walk outside in patients with stroke. Fulk et al. suggested that a comfortable walking speed of 0.93 m/s was the cutoff point indicating limited community and full community ambulators [24]. Although almost all patients in the present study were defined as full community ambulators by walking speed [24], they did not reach the recommended level of MVPA. Thus, the factors associated with MVPA might differ from those with ambulatory discriminations because the components of MVPA were not limited to outside activities. Regardless, it is important to guide patients by emphasizing the importance of MVPA to make sure that they will continue physical activity in their community setting.

### 4.3. Associations between Perceived Built Environment Attributes and Objectively Measured Physical Activity

Although some reports investigated the correlation between built environment attributes and cardiovascular disease and stroke incidence [32,33], few reports from around the world have investigated the correlation between perceived built environment attributes and physical activity after such diseases [16,17,34].

This study is, to our knowledge, the first to investigate the associations of perceived built environment attributes with objectively measured physical activity in patients with stroke. The present results may be useful in providing an appropriate approach to the promotion of physical activity in patients with stroke depending on individual built environment attributes. For instance, we can suggest that patients with stroke who perceive the importance of the presence of sidewalks or accessibility to recreational facilities should walk outside as much as possible to promote their physical activity. The association of physical activity with built environment attributes in community-dwelling patients with stroke partly concurred with the opinion of adults or elderly populations. For example, the average number of steps taken in the present results was significantly associated with the presence of sidewalks consistent with the feelings of Japanese adults as reported by Inoue et al. [9]. They indicated that residential density, access to shops, and the presence of sidewalks were related to walking 150 min/week or more and that access to shops and the presence of bike lanes were related to a high level of MVPA [9]. With regard to the purpose of physical activity, purposes of walking are divided into walking as transportation and walking as recreation. Inoue et al. also reported that social environment and aesthetics consistently correlated with both walking for transportation and walking for recreation among elderly Japanese [11]. Bike lanes, access to recreational facilities, and household motor vehicles were also related to walking for transportation. Shigematsu et al. suggested that elderly adults might combine walking for transportation with that for recreation in one trip [35]. Although the average number of steps taken in the present study was significantly associated with the presence of access to recreational facilities, we could not determine the purpose of the physical activity because the questionnaire did not address this. We also did not investigate marital status and the patient’s role at home or employment status, which also may influence the purpose of walking and physical activity. In any case, as an approach to individual patients with stroke, the first step may be to recommend walking to travel to destinations related to daily life.

Although MVPA was not significantly associated with the perceived built environment attributes in the present study (social environment: β = 0.224, *p* = 0.059), there appeared to be differences in the association between environmental characteristics and the types of physical activity performed, inconsistent with the findings of a previous study [12]. Saito et al. indicated that MVPA performed during leisure time was associated with access to recreational facilities and the number of motor vehicles [12], whereas walking for transportation was associated with the number of motor vehicles, access to shops, and the presence of sidewalks. Additional study is required to elucidate the association between environmental characteristics and types of physical activity such as number of steps taken, the duration of physical activity by intensity, and sedentary behavior.

### 4.4. Limitations

This study has some limitations. First, as the sample population was small, we could not compare age or sex differences of association objectively measured physical activity and built environment attributes. These differences may affect physical activity because the relation between physical activity and environmental factors differ among population groups because of different roles in society. Second, we could not evaluate the patients’ socioeconomic factors such as education level, family support, and family income, which may also influence both the physical activity of stroke patients and built environment attributes or stroke mortality [36,37]. Finally, a cross-sectional investigation can only evaluate the relation between objectively measured physical activity outcomes and perceived built environment attributes. A longitudinal intervention study would be required to verify causal relations. Because the determinants of physical activity are associated with multiple factors [8], future studies may be needed to comprehensively evaluate post-stroke physical activity. However, the present results support the generalizability of our findings on post-stroke physical activity environments because our sample only included community-dwelling ambulatory patients with stroke.

## 5. Conclusions

The present study indicates that the average number of steps were significantly associated with perceived built environment attributes such as the presence of a sidewalk and access to recreational facilities in community-dwelling ambulatory patients with stroke. The consideration of individual built environment attributes might be useful in suggesting a strategy that promotes appropriate physical activity for patients with stroke.

## Figures and Tables

**Table 1:** Clinical characteristics.

Characteristic	All Participants (*n* = 61)
Age (years)	67.0 (55.0–74.0)
Sex (male), n (%)	46 (75.4)
Body mass index (kg/m^2^)	23.2 (22.0–24.8)
Subtypes, n (%)	
Ischemic	48 (78.9)
Hemorrhage	13 (21.3)
NIHSS (score)	1.0 (0–1.0)
Time since stroke (months)	4.0 (3.5–5.2)
Comorbidity, n (%)	
Hypertension	46 (75.4)
Diabetes mellitus	21 (34.4)
Dyslipidemia	35 (57.4)
Walking speed (m/sec)	1.2 (1.0–1.3)
Walk with walking aids	11 (18.0)
Number of steps (/day)	5556.7 (3965.0–7471.3)
MVPA (min/day)	13.9 (3.4–30.3)
MVPA (min/week)	97.0 (24–212)

Abbreviations: NIHSS, National Institutes of Health Stroke Scale; MVPA, moderate-to-vigorous physical activity. Values are shown as median (interquartile range) or as ordinal variables and counts (%) for categorical variables.

**Table 2:** Multivariate regression analysis for physical activity outcomes.

Variable	Number of Steps (/day) (Adjusted R^2^ = 0.33)	
	Β	*p* Value
Age	−0.076	0.503
Sex	−0.185	0.092
NIHSS	−0.101	0.406
Walking speed	0.347	0.012
Sidewalks	0.274	0.016
Access to recreational facilities	0.284	0.010
**Variable**	**MVPA (min/day)** **(Adjusted R^2^ = 0.21)**	
	**Β**	***p* Value**
Age	0.150	0.232
Sex	−0.200	0.105
NIHSS	−0.024	0.858
Walking speed	0.405	0.008
Residential density	0.082	0.508
Sidewalks	0.112	0.356
Social environment	0.224	0.059

Abbreviations: NIHSS, National Institutes of Health Stroke Scale; MVPA, moderate-to-vigorous physical activity.
